# Protective effects of exosomes derived from lyophilized porcine liver against acetaminophen damage on HepG2 cells

**DOI:** 10.1186/s12906-021-03476-y

**Published:** 2021-12-18

**Authors:** Riccardo Tassinari, Claudia Cavallini, Elena Olivi, Valentina Taglioli, Chiara Zannini, Orlando Ferroni, Carlo Ventura

**Affiliations:** 1 National Laboratory of Molecular Biology and Stem Cell Bioengineering of the National Institute of Biostructures and Biosystems (NIBB), at the Innovation Accelerator, CNR, Via Piero Gobetti 101, 40129 Bologna, Italy; 2 Neorland Srl, Via Del Sale, 40/A, 26100 Cremona, Italy

**Keywords:** Extracellular vesicles, Porcine liver exosomes, Liver hepatocytes, Nutraceuticals, Preconditioning

## Abstract

**Background:**

Recently, extracellular vesicles have come to the fore following their emerging role in cell communication, thanks to their ability to reach cells into the human body without dissipating their cargo, transferring biological active molecules, such as proteins, nucleic acids, lipids, etc. They appear as a promising tool in medicine, because of their capability to modulate cellular response in recipient cells. Moreover, a considerable number of publications suggests that exosome uptake is selective but not specific, and it can cross species and cell-type boundaries. This study aims to explore the potential role of porcine liver derived extracellular vesicles, exosomes in particular, to protect human cells from acute damage induced by acetaminophen.

**Methods:**

Extracellular vesicles were isolated from porcine lyophilized liver using polymer-based precipitation and a further enrichment was performed using affinity beads. The effects of obtained fractions, total extracellular vesicles and enriched extracellular vesicles, were assessed on human liver derived HepG2 cells. Cell growth and survival were tested, with MTT and area coverage analysis designed by us, as well as protein expression, with immunofluorescence and Western blot. Oxidative stress in live cells was also measured with fluorogenic probes.

**Results:**

After proving that porcine extracellular vesicles did not have a toxic effect on HepG2, quite the contrary total extracellular vesicle fraction improved cell growth, we investigated their protective capability with a preconditioning strategy in APAP-induced damage. EVs displayed not only the ability to strongly modulate cell survival responses, but they also were able to boost cell cycle progression.

**Conclusions:**

Extracellular vesicles derived from farm animal food derivatives are able to modulate human hepatic cell metabolism, also improving cell survival in a damaged context.

**Supplementary Information:**

The online version contains supplementary material available at 10.1186/s12906-021-03476-y.

## Background

In recent years, the prominent role of extracellular vesicles (EVs) in cell-cell communication is emerging. In addition to well-known naked molecules, cells release vesicles into the extracellular environment in a fine-tuned fashion, dependent upon cell type as well as metabolic state of the cell itself. These vesicles are usually referred to as microvesicles, ectosomes, shedding vesicles, or microparticles, depending on different factors, such as their origin, dimension or membrane features [1]. EVs are defined as particles released from the cell and delimited by a lipid bilayer, that can be additionally divided in two subtypes: one with an endosome origin, formed by “exosomes”, and one derived from plasma membrane bubbling, formed by “ectosomes” (microparticles/microvesicles). Therefore, exosomes are nanoparticles, with a diameter ranging from 40 to 160 nm, generated by multivesicular late endocytic compartments (multivesicular bodies (MVBs)) and secreted by fusion of MVBs with plasma membrane. In order to distinguish exosomes from different EVs, diversified approaches have been used: first of all, physical characteristics of EVs, but also biochemical composition of their membrane (CD63+/CD81+, etc.) and/or of their cargo [2].

Besides a common set of molecules, such as heat shock proteins Hsp70 and Hsp90, endosome-associated proteins (Rab5, annexin, etc.), as well as proteins of tetraspanin family (CD9, CD63, CD81, CD82, etc.) that contribute to their structure and function, exosomes contain specific components, determined by their parental cell origin [1]. Definite composition of exosome cargo suggests the existence of a specific mechanism that controls molecule sorting, and, moreover, different pathways seem to be involved in this process [3].

An exosome intriguing feature consists in their pleiotropic effect: exosomes derived from a specific cell type can act on different cell types, as demonstrated by hepatoprotective effect of mesenchymal stromal cell-derived exosomes [4], but interspecies exchanges have also been reported. Recent studies demonstrated that exosomes naturally present in cow milk could be up-taken by human cancer cell lines and by rodents, as proved by in vivo experiments [5], as well as murine cell exosomes are able to transfer their content in human recipient mast cells [6].

In the human body, the largest internal organ is the liver, which has a crucial role in many physiological and pathological processes, being involved in metabolism, detoxification, digestion, synthesis and storage [7]*.* Hepatocytes account for 80% of organ cellularity, and regulate almost all liver functions, such as glucose and lipid metabolism and partitioning, plasma protein and bile acid synthesis, as well as detoxification and regeneration [8]. Hepatocytes are fully differentiated parenchymal cells, with a remarkable and unique capacity to re-enter into the cell cycle, proliferate and restore functional liver mass, with stem-like proliferative capabilities [9].

Recent findings demonstrate how exosomes may play an active role in pathological conditions, such as drug induced liver injury (DILI). Exosomes are recognized as sensitive and specific biomarkers, as shown, for example, by increased release of exosomal miRNA-122 in human primary hepatocytes treated with tolvaptan drug [10]. Intriguingly, it is now clear that exosomes are not merely signals of some kind of injury, but they are actively involved in organ repair and regeneration, also in the liver. For instance, murine hepatocyte-derived exosomes are able to restore liver function in an animal model of liver ischemia/reperfusion injury, through delivering of sphingosine-1-phosphate into recipient cells [11].

Acetaminophen (APAP) is a largely used antipyretic and analgesic drug that could have dramatic effects in over-dosage, causing acute failure damage. Acetaminophen acts as a dose-dependent hepatotoxin, that can cause severe acute hepatocellular injury [12] inducing oxidative stress, apoptosis and necrosis, and its overdose is one of the most common causes of DILI i Western countries [13]. Human hepatoma cell line HepG2 is largely used in studies on drug hepatotoxicity and metabolism, also regarding acetaminophen toxicity [14].

To assess the effect of farm animal food derivatives on human cells, which may be used as nutraceuticals, commercially available lyophilized porcine liver Neorland® Epatoguna (Guna, Milan) was tested, under the assumption that the patented industrial process should have no or little impact on EV content and integrity. We investigated if porcine liver derived exosomes, isolated from young and healthy animals, can have an impact on human liver cells, both in physiological and pathological conditions.

## Methods

### Porcine liver extract

Lyophilized porcine livers were obtained from Neorland s.r.l. (Cremona, CR, Italy). Livers were isolated from young swine immediately after slaughtering,from healthy pigs (both male and female) under the 150 kg of weight. The slaughtering process complies with the requirements of EU Regulation 852/04 and 853/04, and animal welfare was ensured by compliance with EU Regulation 2073/05 and 1099/09. Briefly, pigs of Italian origin were selected according to young age and perfect state of health. After removal, livers were wrapped in food storage containers to be sent to the freezing phase. After freezing at − 20 °C, the liver was subjected to a sublimation process, following which all remaining water was removed by drying. The final product was obtained by grinding, and, after excluding the presence of impurities, sealed in a triple layer aluminum bag.

### Cells and reagents

Human hepatocyte carcinoma HepG2 cells (Sigma-Aldrich, St. Louis, MO, USA) were cultured in EMEM (Lonza, Walkersville, MD USA) containing 1% non-essential Amino Acids, 2 mM L-Glutamine (Lonza), 0.5% penicillin-streptomycin solution (Lonza) and 10% fetal bovine serum (FBS) (Gibco, Thermo Fisher Scientific, Carlsbad, CA, USA), and maintained in 5% CO_2_ humidified atmosphere at 37 °C. Sub-confluent cultures (70-80%) were split from 1:3 to 1:6, using 0.25% trypsin/EDTA (Lonza). Medium was changed every 2 days. To perform experiments, cells were seeded at 2-3 × 10^4^ cells/cm^2^ and, after 24 h, the medium was replaced with an exosome-free complete medium, obtained by replacing FBS with exosome-depleted FBS (Gibco). After thawing, cells were used from passage 2 to 8. To induce DILI, cells were treated with APAP (Sigma-Aldrich). All cell culture plasticware were purchased from Corning (Corning, Glendale, AZ, USA).

### Exosome isolation, purification and quantification

One gram of lyophilized liver was resuspended in 10 ml of Krebs-Ringer Bicarbonate Buffer (Sigma-Aldrich) additioned with CaCl_2_ 3 mM (Sigma-Aldrich), and stirred for 1 h in constant shaking to facilitate solubilization. After that, the solution was transferred to the incubator at 37 °C, and 0.5% Collagenase IA (Sigma-Aldrich) was added. After 1 h, the solution was removed from the incubator and put at 4 °C in an ice-water bath to stop digestion. To remove debris, scalar filtrations were performed, starting with CD-1™, Cell Dissociation Sieve - Tissue Grinder Kit (Sigma-Aldrich), and continuing with cell strainer from 100 μm to 40 μm pore size (Corning). At the end, the solution was eluted to syringe filters, with a pore size ranging from 5 μm to 0.2 μm (Sartorius Stedim Biotech GmbH, Goettingen, Germany), and centrifuged at 1500 x g for 30 min. Supernatant was removed, mixed with ExoQuick solution (System Biosciences, Mountain View, CA), and incubated at 4 °C overnight. Next day, total EVs were pelleted (1500 x g for 30 min), supernatant was removed, and EVs were resuspended in 300 μl of PBS buffer. One hundred and fifty μl of total EVs were further enriched using Exo-Flow magnetic beads (System Biosciences). Briefly, magnetic beads were coupled with anti-Rab5b antibody, and incubated with total EVs overnight on a rotating rack at 4 °C. Afterwards, enriched exosomes were eluted from magnetic beads and resuspended in 300 μl of Exosome Elution Buffer. The first fraction obtained was named total EV (TEV) fraction while the second one was called enriched EV (EEV) fraction.

### Sample normalization with ELISA

Porcine CD81 antigen (CD81) ELISA Kit was purchased from MyBioSource (San Diego, CA, USA). Data were obtained with protocol according to the manufacturer’s instructions. Quantitative data obtained from ELISA were used exclusively to normalize EEV and TEV samples. EEV fraction was diluted to have CD81 final concentration of 0.1 ng/ml. To make comparable the number of exosomes contained in both fractions, also the TEV fraction was diluted the same number of times as the enriched fraction.

### Exosome uptake in HepG2

To visualize EV uptake, vesicles were stained with BODIPY™ TR Ceramide (Molecular Probes, Invitrogen, Thermo Fisher Scientific), following the manufacturer’s instructions. One hundred μl of EV samples, resuspended in PBS buffer, were incubated in a solution with a final dye concentration of 1-10 mM for 20 min at 37 °C. As negative control for background staining in uptake assays, 1 mM BODIPY™ TR Ceramide was added to 100 μl of PBS without exosomes. Excess unincorporated dye from labeled exosomes was removed with Exosome Spin Columns (MW 3000; Invitrogen), following the manufacturing protocol. BODIPY™ TR Ceramide-stained exosomes were added to receiving cells in Exo-free completed medium. Cells were visualized after 3 h and 24 h post-treatment with Nikon Inverted Microscope Eclipse Ti-E equipped with a Digital Sight camera DS-Qi2Mc (Nikon Instruments, Tokyo, Japan). Images were analyzed with NIS-Elements (Nikon Instruments) and at least 3 independent experiments were performed for each point.

### Cell growth and viability assays

Cell number was evaluated through microscope images or MTT assay. Due to the difficulty to separate and count single HepG2 cells, we have identified a strategy to evaluate the amount of covered area, which we verifying to be proportional to cell number (Additional file 1). Cells were seeded in 48-well plates, treated with or without TEV or EEV fractions and with or without APAP, and photographed after 0, 24, 48 and 72 h. Percentage of uncovered areas was registered every day. Ratio between uncovered area at a given day and the uncovered area at day zero was calculated. Calculated values are inversely proportional to growing rate. MTT [3-(4,5-dimethylthiazol-2-yl)-2,5-diphenyltetrazolium bromide] reduction assay was performed as recommended by the manufacturer (Sigma-Aldrich). HepG2 were seeded into 24-well plates and treated with or without EEV or TEV fraction and with or without APAP. At the end of treatments, cells were incubated with MTT at the concentration of 0.5 mg/ml. After 2 h at 37 °C in a CO_2_ incubator, formed formazan crystals were dissolved with 200 μl per well of dimethylsulfoxide (DMSO, Sigma-Aldrich). Absorbance was measured at 570 nm (Multiskan™ FC Microplate Photometer, Thermo Fisher Scientific). At least 3 independent experiments were performed for each data set.

### Western blot

After specific treatments, cells were lysed using Mammalian Protein Extraction Reagent (M-PER, Thermo Fisher Scientific) containing protease and phosphate inhibitors (Sigma-Aldrich). After lysis, protein concentrations were measured using Bradford Reagent (VWR International, Radnor, Pennsylvania, USA). Cell lysates were resuspended in Laemmly buffer (Bio-Rad, Hercules, CA, USA) and boiled for 5 min. Fifteen μg of cell lysates were separated by SDS-PAGE on a 10% Stain-Free™ Precast Protein Gel (Bio-Rad) and transferred to a 0.2-mm nitrocellulose membrane (Bio-Rad) with Trans-Blot® Turbo™ Transfer System (Bio-Rad). After blocking, nitrocellulose membrane was incubated with primary antibody solution overnight at 4 °C in constant shaking. The next day, the membrane was washed and probed with horseradish peroxidase (HRP) conjugated secondary antibody (1:10,000 dilution; Cell Signaling Technology, Danvers, MA, USA) for 1 h. Bound antibodies were detected with the use of Clarity Western ECL Substrate (Bio-Rad) and quantified by densitometry with ChemiDoc Touch™ Imaging System (Bio-Rad). For each sample, detected signal was normalized to the total protein amount determined with stain-free acquisition. Primary antibodies used for Western analysis (1:2000 dilution) were all purchased by Cell Signaling Technology: cleaved Caspase-3 (c-Casp3, #9664), cleaved PARP (c-PARP, #5625), Phospho-SAPK/JNK (p-JNK, #4668), Phospho-c-Jun (p-c-Jun, #3270), BiP (#3177). As secondary antibody, 1:10,000 anti-rabbit IgG, HRP-linked Antibody (#7074) (Cell Signaling Technology) was used.

### Immunofluorescence

After specific treatments, HepG2, grown on glass coverslips in 24-well plates (VWR International), were fixed with neutral buffered formaldehyde (VWR International) at room temperature (RT) for 10 min. After this, cells were washed with PBS/0.25% Tween 20 (Sigma-Aldrich) and permeabilized with 1% Triton X-100 (Sigma-Aldrich) at RT for 10 min. Incubation with 4% bovine serum albumin (BSA) (Sigma-Aldrich) in PBS for 1 h at RT was performed to prevent aspecific antibody binding. Primary antibodies were added overnight at 4 °C in a humid chamber, and the next day, after 3 washes, cells were stained at RT for 30 min with secondary antibodies. Nuclei were counterstained with NucBlue™ Fixed Cell ReadyProbes™ Reagent (DAPI) (Molecular Probes, Invitrogen). Samples were mounted with ProLong Gold Antifade Mountant (Thermo Fisher Scientific). Images were acquired with a Digital Sight camera DS-Qi2Mc (Nikon Instruments) through the imaging software NIS-Elements (Nikon Instruments).

The primary antibodies used (1:200 dilution) were as follows: Ki67 (#TA336566, Origene, Rockville, Maryland, USA), p53 (TP53) (#TA502870, Origene), p-p53 (Phospho-Ser15) (#TA326166, Origene), p21 (#sc397, Santa Cruz Biotechnology, Dallas, Texas, USA), p27 (#sc1641, Santa Cruz Biotechnology). The secondary antibodies used (1:1000) were: goat anti-rabbit IgG (H + L) highly cross-adsorbed, Alexa Fluor Plus 488 (#A32731, Invitrogen), and goat anti-mouse IgG (H + L) highly cross-adsorbed, Alexa Fluor Plus 555 (#A32727, Invitrogen).

### In vivo imaging of oxidative stress metabolic markers

To assess oxidative stress, cells were cultured in 24-well plates and treated with or without APAP and in presence or absence of EV fractions. Different oxidative markers (purchased from Thermo Fisher Scientific) were evaluated: CellROX™ Orange Reagent, MitoTracker™ Green FM and MitoSOX™ Red Mitochondrial Superoxide Indicator. CellROX™ Orange Reagent was added to the cells at a final concentration of 5 μM and incubated for 30 min at 37 °C. Then, cells were washed three times with PBS and visualized with Nikon Inverted Microscope Eclipse Ti-E equipped with a Digital Sight camera DS-Qi2Mc (Nikon Instruments) and images were acquired with NIS-Elements (Nikon Instruments). At least 3 experiments were performed for each data set.

MitoTracker™ was dissolved in high-quality DMSO to a final concentration of 1 mM. For experiments, MitoTracker™ stock solution was diluted in EMEM to the final working concentration (200 nM). Staining solution containing MitoTracker™ was added to cells and incubated for 30 min under growth conditions. Then, staining solution was replaced with fresh prewarmed medium and cells were observed using Nikon Inverted Microscope Eclipse Ti-E equipped with a Digital Sight camera DS-Qi2Mc (Nikon Instruments). Images were analyzed with NIS-Elements (Nikon Instruments) and at least 3 independent experiments were performed for each point.

A reagent stock solution of MitoSOX™ mitochondrial superoxide indicator was prepared by dissolving powder in high-quality DMSO. A 2 μM MitoSOX™ reagent working solution in HBSS/Ca/Mg (Hankʼs balanced salt solution with calcium and magnesium) was added to cells and incubated 10 min at 37 °C and 5% CO_2_, protected from light. Then, cells were washed three times with PBS. Cells were visualized with Nikon Inverted Microscope Eclipse Ti-E equipped with a Digital Sight camera DS-Qi2Mc (Nikon Instruments) and images were acquired with NIS-Elements (Nikon Instruments). At least 3 experiments were performed for each data set.

### Statistical analysis

Data were analyzed using PRISM 7.05 for Windows, (GraphPad Software, La Jolla California, USA). Data was expressed as the mean ± SD. Differences in multiple group comparisons were assessed by a one-way ANOVA followed by Tukey’s multiple comparison test. Significant differences were defined as those with *P* values smaller than 0.05.

## Results

### Exosome isolation, quantification and cellular uptake

EVs, exosomes in particular, from lyophilized porcine liver, were obtained as described in the Methods section, using ExoQuick reagents. TEV fraction isolated from 1 mg of lyophilized porcine liver was resuspended in 300 μl of PBS. A further enrichment (EEV) was obtained from the TEV fraction, using magnetic beads coupled with specific exosomal antibody Rab5. ELISA specific for porcine CD81 was used to make exosome content of TEV and EEV fractions comparable. After verifying that concentration of CD81 calculated with ELISA was proportional to standard exosome dilution (Additional file 2), results obtained for EEV fraction were used to normalize both EEV and TEV. In particular, EEV fraction was diluted at 0.1 ng/ml of CD81, and TEV fraction was diluted as many times as EEV. Concentration of CD81 protein did not provide a quantitative mean in number of exosomes, but was useful to implement a kind of normalization, in order to avoid that a fraction may contain a number of exosomes significantly different from another one.

### EV uptake in HepG2 cells and effect on cell growth

The ability of liver-derived HepG2 human cells to uptake porcine origin particles was assessed with TEV and EEV fractions. EVs were labeled with BODIPY™ TR Ceramide. Cells were seeded at 50% confluence and, after 24 h, treated with or without 1 μl of labeled EEV as well as TEV fraction. The ceramide fluorescence probe could be internalized into recipient cells only through membrane-mediated exosome uptake, and, once internalized and dismantled, it produces a selective staining of the Golgi complex (Additional file 3).

**Fig. 1:**
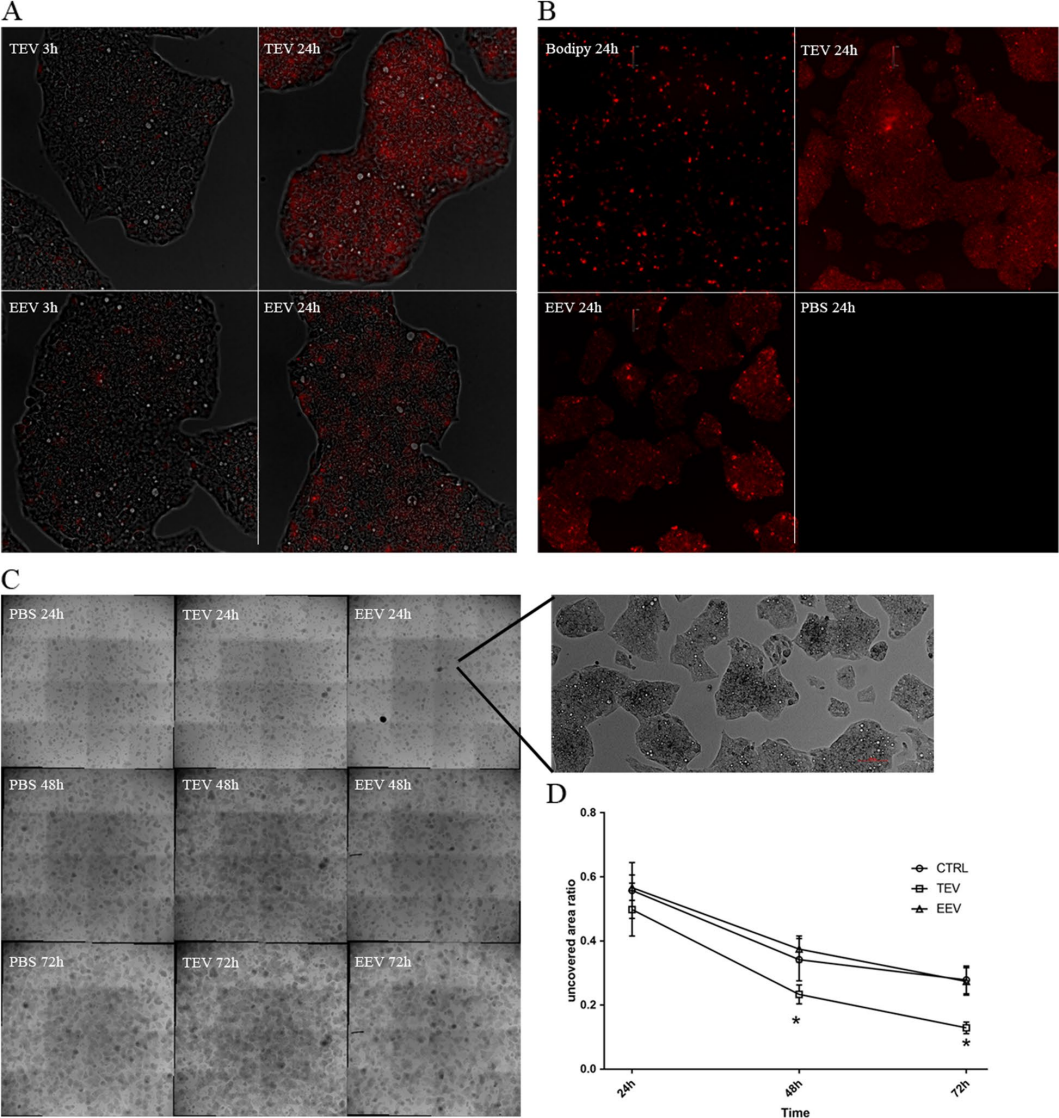
Uptake of TEV and EEV fraction in HepG2 cells and effects on cell growth. **A** BODIPY™ TR Ceramide labelled (red) TEVs and EEVs showing uptake in HepG2 cells at 3 h (left) and 24 h (right). Shown here are microphotographs (384 × 384 μm) taken stitching 4 images of HepG2 cells cultured on a 48-well plate; representative for 5 experiments. **B** BODIPY™ TR Ceramide labelled (red) or unlabelled uptake on HepG2 cells at 24 h. Upper-left: direct BODIPY™ labelling in culture medium of HepG2; upper-right: uptake of BODIPY™ labelled TEVs; bottom-left: uptake of BODIPY™ labelled EEVs; bottom-right: BODIPY™ labelled PBS without vesicles after elution on Exosome Spin Column on HepG2 cells. Shown here are microphotographs (1560 × 1560 μm) taken stitching 4 images of HepG2 cells cultured on a 48-well plate; representative for 3 experiments. **C** Effects of TEV and EEV fractions on HepG2 cell growth. Microphotographs (5800 × 5800 μm) of control cells (PBS, left panels), TEV-treated cells (middle panels) and EEV-treated cells (right panels) at 24, 48 and 72 h. Zoomed on the right, there is a portion of the whole image that shows clearly the cell islets. Red line is 100 μm. **D** Graph representing uncovered area ratio (uncovered area at the mentioned time/uncovered area at day 0) by treated or untreated HepG2 cells over time. Consequently, the lower the ratio, the higher the growth of cells. Areas were calculated after binarization of the images in the C panel. Data are mean ± SD with *n* = 3 per group. *, *p* < 0.05 vs control

After treatment with both stained fractions, we immediately imaged the living cells in order to understand whether, and in the affirmative, after which time vesicles could ingress the cells. Human and porcine receptors for exosomes share a very high amino acid sequence homology, and in fact after only 30 min red spots inside the cells were observable (Additional file 4,), appearing clearly visible after 3 h (Fig. 1A, left panels), and reaching the maximum intensity at 24 h (Fig. 1A, right panels). At 24 h all the cells appeared dyed, and while inside the cell, the BODIPY™ TR Ceramide was virtually all complexed inside the Golgi, the natural cellular compartment of affinity, suggesting that the microvesicles have been dismantled and processed, freeing the fluorescent dye. In order to support this hypothesis, two kinds of negative control were performed. Two solutions of PBS with BODIPY™ TR Ceramide and without exosomes were prepared. One of these, used as dye-only control, was directly added in the culture medium. The other one, used as negative control, was eluted through the Exosome Spin Column. In the dye-only control group, a weak fluorescent signal was detectable (Fig. 1B, upper-left), but fluorescence appearance was totally different. Dye-only control demonstrated that fluorescence detected inside EEV- and TEV-treated cells was not due to dye contamination. In contrast, in the negative control of PBS without exosomes no fluorescence was detected inside the cells (Fig. 1B, bottom-right), confirming that elution through Exosome Spin Column was able to completely eliminate free dye and, even more relevant, the only way by which BODIPY™ TR Ceramide can enter cells is after the incorporation in the phospholipid layer of the vesicles, if present.

While we confirmed the biodisponibility of the TEV and EEV fractions, we checked their effect on HepG2 in terms of growth. Due to the fact that cells were prone to cluster together, affecting cell count, and it was almost impossible to disaggregate them without creating any type of damage, we decided to better quantify cell by growth measuring the total area coverage on the cell culture plate. The analysis was performed on 48-well plates at 24, 48 and 72 h, with cells treated or untreated with TEV and EEV fractions (Fig. 1C). As shown in Fig. 1D, TEV fraction increased cell growth significantly compared to control cells, and this effect was evident after 48 h, increasing until 72 h. In contrast, no difference between control cells and EEV fraction-treated cells was detected.

### Protection from acetaminophen-induced damage

Effects of human derived exosomes on different cell types are largely investigated and their possible roles in therapy are deeply explored. This paper aims to investigate a possible protective effect of porcine derived EVs on human cells. Thus, we introduced an APAP treatment to assess the ability of the TEV and EEV fractions to counteract the drug-induced damage. APAP is toxic for HepG2 in a dose-dependent manner, and results in cell death. The effects of APAP alone were evaluated in a first set of experiments throughout MTT assay and nuclei count, and used on HepG2 at 10 mM.

**Fig. 2:**
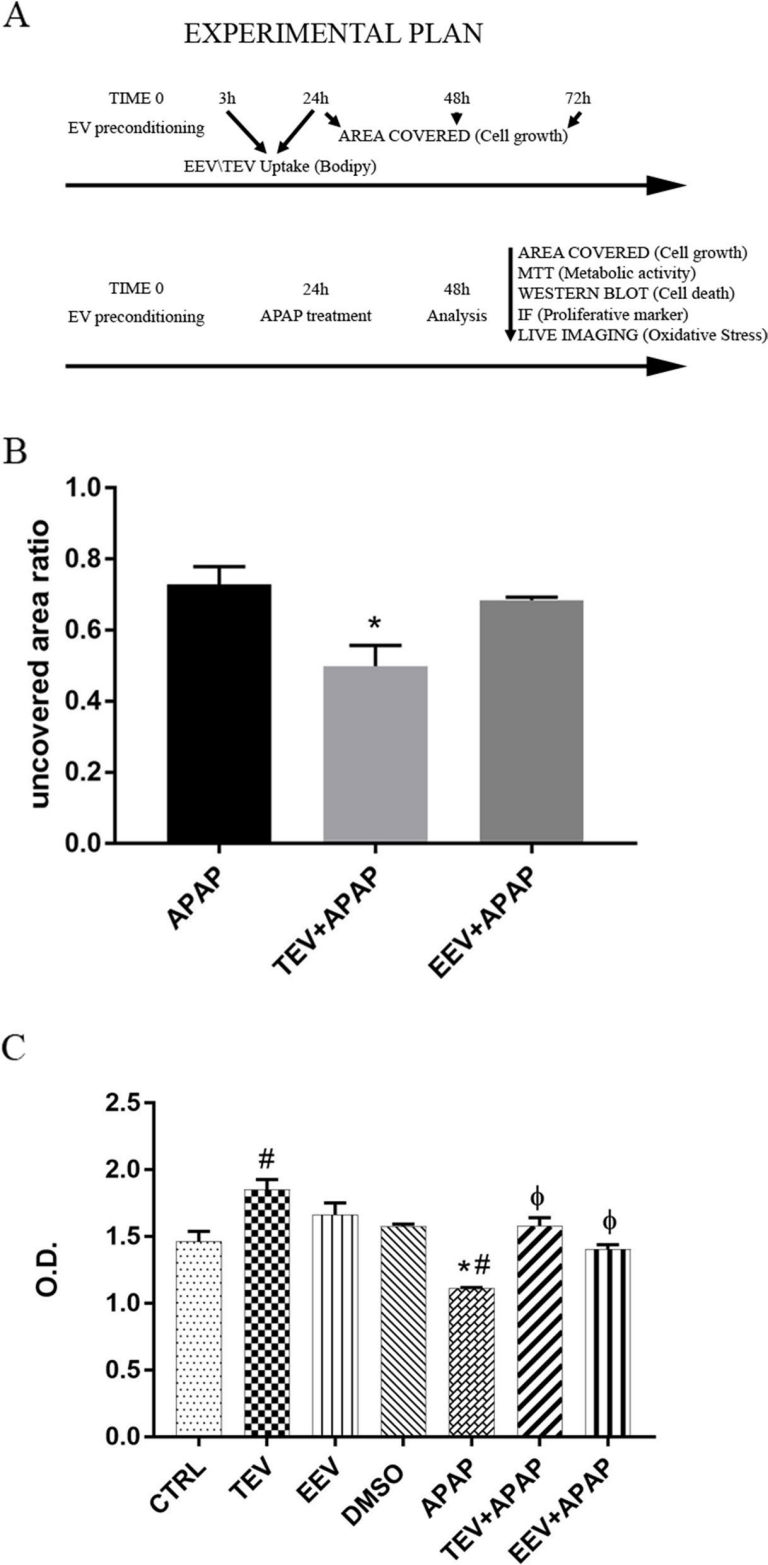
Evaluation of protection activity mediated by EVs after APAP-induced damage. **A** Experimental outline of the preconditioning strategy implemented to test EV-pretreatment protective effects. Shown in the first diagram the experiments timeline performed after precondition with EV and without APAP treatment. Shown in the second diagram, 24 h after EV preconditioning, cells were treated with or without APAP; after further 24 h analyses were performed. **B** TEV-pretreatment improved cell survival on HepG2 cells treated with APAP. Diagram showing uncovered area ratio: uncovered area after 24 h with (EEV and TEV) or without (CTRL) EVs and subsequent 24 h of APAP/uncovered area at day 0. The total loss of HepG2 cells is calculated after binarization of cell absence/presence in cultured HepG2 cells prior and after APAP treatment. **C** MTT activity detected in HepG2 cells with or without EV treatment and with or without APAP administration. The 4 samples starting from the left of the diagram did not receive APAP treatment, i.e. control cells (CTRL), TEV- and EEV-treated cells (TEV, EEV), vehicle-treated cells (DMSO), conversely the last 3 did receive it: cells treated with APAP alone (APAP), or pretreated with TEV and EEV fractions (TEV + APAP, EEV + APAP). As shown, TEV treatment was able to induce an increase in detectable mitochondrial activity in all conditions, meanwhile EEVs displayed an effect only in APAP-treated samples. For all panels, data are mean ± SD with *n* = 3 per group. *, *p* < 0.05 vs CTRL; #, *p* < 0.05 vs vehicle (DMSO); Φ, *p* < 0.05 vs APAP

Once verified that APAP concentration was able to severely affect cell viability, we tested the protective effects of porcine liver derived EVs on cells with a preconditioning strategy. Nanovesicles were added to cells in a medium supplemented with 10% exosome-free FBS for 24 h, after which APAP was administered. Area coverage and MTT analysis were performed after additional 24 h (Fig. 2A). As shown in Fig. 2B in presence of APAP, the treatment with TEV fraction mitigated cell death, allowing cells to grow during the next 24 h. This result was confirmed by MTT analysis (Fig. 2C). Even if both analyses displayed convergent data underlying the protective effect of TEV fraction on HepG2 cells, there was a slight difference in quantitative data. Actually, while the protective effect of TEV fraction on cells treated with APAP was significant in both kinds of analysis, only with MTT assay EEV fraction seemed to have this capability. We speculated that this observation may result from the different parameters measured by the two assays. Coverage area assay measured cell number, while in MTT metabolism rate was detected, mitochondrial metabolism in particular. It is very likely that two effects combined: first, a higher metabolism rate per cell, and second an increased total cell number, thus suggesting a proliferative effect on the HepG2 cells.

### Evaluation of cell cycle and metabolic markers

**Fig. 3:**
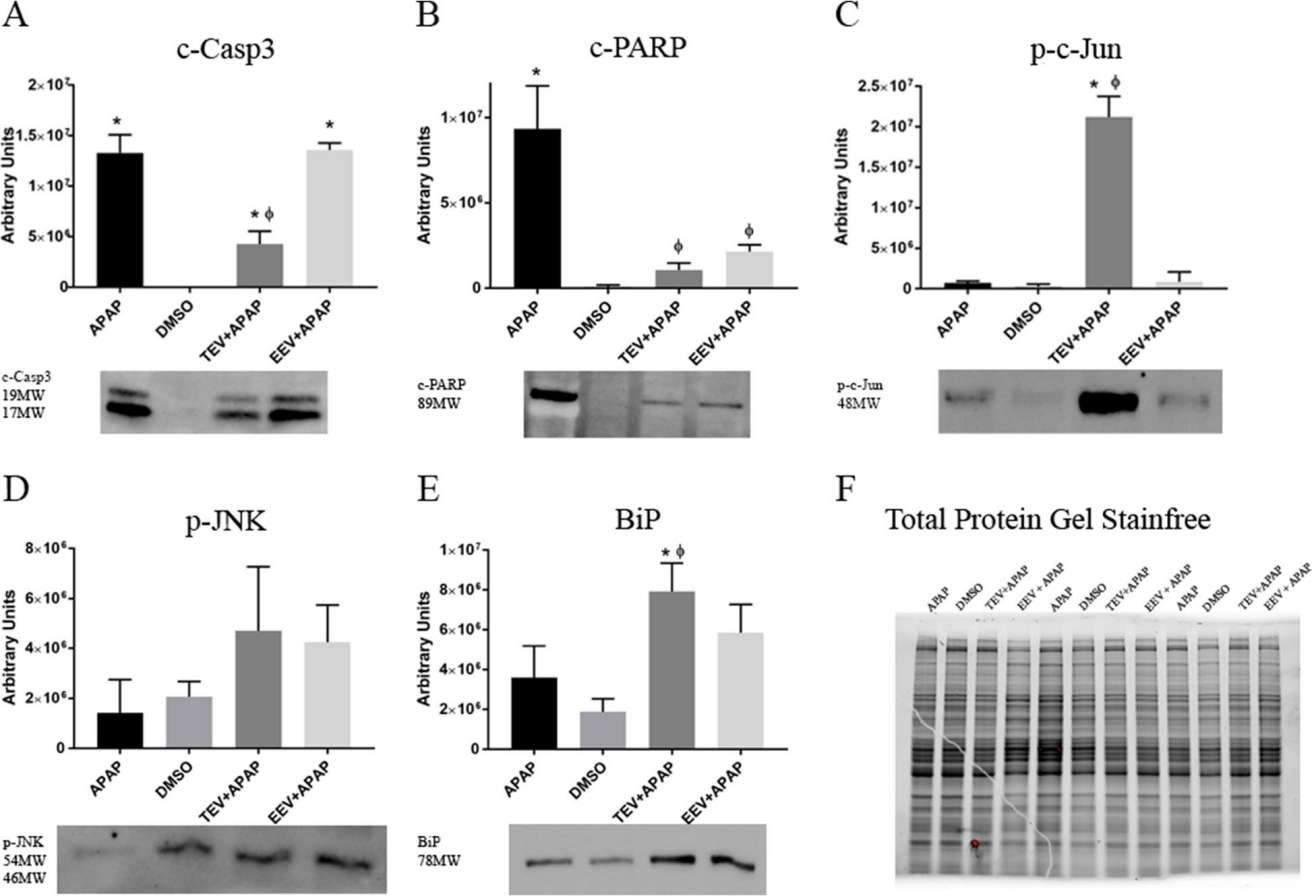
Evaluation of metabolic marker proteins: TEV and EEV treatments differently affected protein expression after APAP administration. Diagrams showing densitometric analysis of protein levels (**A**, c-Casp3; **B**, c-PARP; **C**, p-c-Jun; **D**, p-JNK; **E**, BiP) in HepG2 APAP-treated cells, vehicle-treated cells (DMSO) and APAP plus TEV or EEV pretreatments. All protein levels are normalized to the total protein expression of each sample measured using the TGX stain-free technology by Bio-Rad (**F**). For all panels, data are mean ± SD with *n* = 3 per group. *, *p* < 0.05 vs DMSO (control); Φ, *p* < 0.05 vs APAP. (Shown lines were cropped from the original row file. Full-length blots are displayed in (Additional file 5)

To investigate the contribution of each pathway, the improvement of metabolic parameters and/or the higher proliferation rate, we decided to analyze selected protein expression. In a first set of experiments, Western blot analyses of stress hallmark proteins were performed (Fig. 3). As confirmation of cell mortality reduction in cells pretreated with TEV fraction, we found a decrease, compared to cells treated with APAP alone, in the cleaved Caspase-3, which was not detectable in EEV fraction-treated cells (Fig. 3A). On the other hand, both EEV and TEV fractions were able to turn down c-PARP (Fig. 3B), the activated isoform of PARP, cleaved by Caspase during apoptosis. Intriguingly, TEV fraction was able to induce an overexpression of phosphorylated c-Jun, that is strongly involved in cell cycle progression. TEV and EEV fractions were able to slightly increase the phosphorylation of c-Jun N-terminal kinase JNK, although in a non-significant manner (Fig. 3D). In the same way of p-JNK, another protein involved in endoplasmic reticulum (ER) stress response, BiP, showed a modulation, being strongly enhanced by the TEV fraction. Adipogenic proteins involved in hepatic lipid metabolism as well as in pathological cellular steatosis were also analyzed, showing no significant modulations (Additional file 6).

**Fig. 4:**
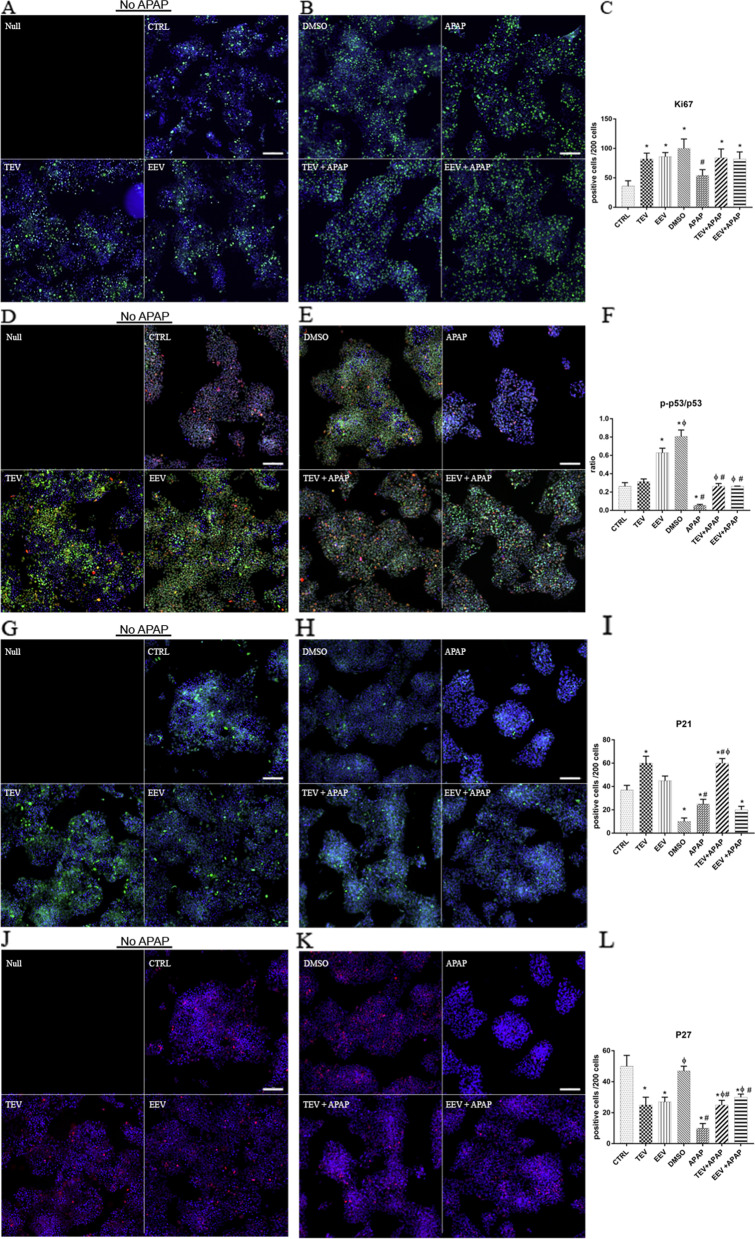
Immunofluorescence of proteins involved in cell cycle progression and apoptosis. Microphotographs taken stitching 4 images of HepG2 cells cultured on a glass coverslip of a PFA-fixed immunofluorescence, representative for 3 experiments (scale bar = 50 μm). Without APAP (panels **A**, **D**, **G**, **J**): no staining (Null), control cells (CTRL), TEV- and EEV-treated cells. With APAP (panels B,E,H,K): vehicle-treated cells (DMSO), control cells (CTRL), TEV- and EEV-treated cells. **A-B** Ki67 staining (green) and DAPI counterstaining (blue) of HepG2 cells. **C** Diagram showing number of Ki67 positive cells in CTRL, TEV- and EEV-treated cells with or without 24 h APAP (or vehicle) treatment. Counting of Ki67 positive nuclei was performed in 3 different fields of view after reaching 200 counted nuclei. **D-E** p-p53 staining (green), p53 staining (red) and DAPI counterstaining (blue) of HepG2 cells. **F** Diagram showing the ratio between p-p53 and p53 positive cells. Counting of p-p53 or p53 positive nuclei was performed in 3 different fields of view after reaching 200 counted nuclei. **G**-**H** p21 staining (green) and DAPI counterstaining (blue) of HepG2 cells. **I** Diagram showing number of p21 positive cells. **J**-**K** p27 staining (red) and DAPI counterstaining (blue) of HepG2 cells. **L** Diagram showing number of p27 positive cells. For all panels, data are mean ± SD with *n* = 3 per group. *, *p* < 0.05 vs CTRL, Φ *p* < 0.05 vs APAP treatment, #, *p* < 0.05 vs vehicle (DMSO)

Data obtained by Western blot suggested that TEV and EEV fractions mediated an anti-apoptotic effect on cells, combined with an impetus in cell cycle progression and a modest increase in protein involved in stress responses. A deeper investigation of different identified pathways was performed with immunofluorescence, as well as in vivo live imaging. Immunofluorescence of Ki67 (Fig. 4A-C), a protein strictly involved in cell cycle progression, was strongly affected by TEV and EEV fraction administration. As shown in Fig. 4A, Ki67 was upregulated in EV-treated cells with respect to control cells, without any significant difference between TEV and EEV groups. APAP did not affect Ki67 expression, even though its vehicle alone, DMSO in low concentration, as expected, enhanced Ki67 proliferation marker (Fig. 4B). Even in the presence of APAP, TEVs and EEVs were still able to elicit an upregulation of Ki67 expression of similar magnitude as that they induced in the absence of the hepatotoxic agent (Fig. 4B).

The observed upregulation of PARP inspired us to visualize its downstream proteins, p-p53 (Fig. 4D-F) and p21 (Fig. 4G-I). Unexpectedly, both proteins were upregulated in EV-treated cells in the presence of APAP, as compared with APAP alone (Fig. 4E, H). In particular, both TEV and EEV preconditioning resulted in increased p-p53 expression in APAP-treated HepG2 cells, when compared to the protein levels detected in cells solely exposed to APAP (Fig. 4E). On the other hand, only TEV preconditioning enhanced p21 expression in the presence of APAP, as compared to APAP-treated cells (Fig. 4H). In the absence of APAP, analysis of protein modulation revealed a significant raise only in p-p53 or p21 in EEV- or TEV-preconditioned, respectively (Fig. 4D, G). Following APAP exposure, p27 expression followed a similar behavior in both TEV- and EEV-preconditioned cells, but it was conversely downregulated, in comparison to untreated controls, in cells that received TEV or EEV in the absence of a subsequent APAP exposure (Fig. 4J-L).

**Fig. 5:**
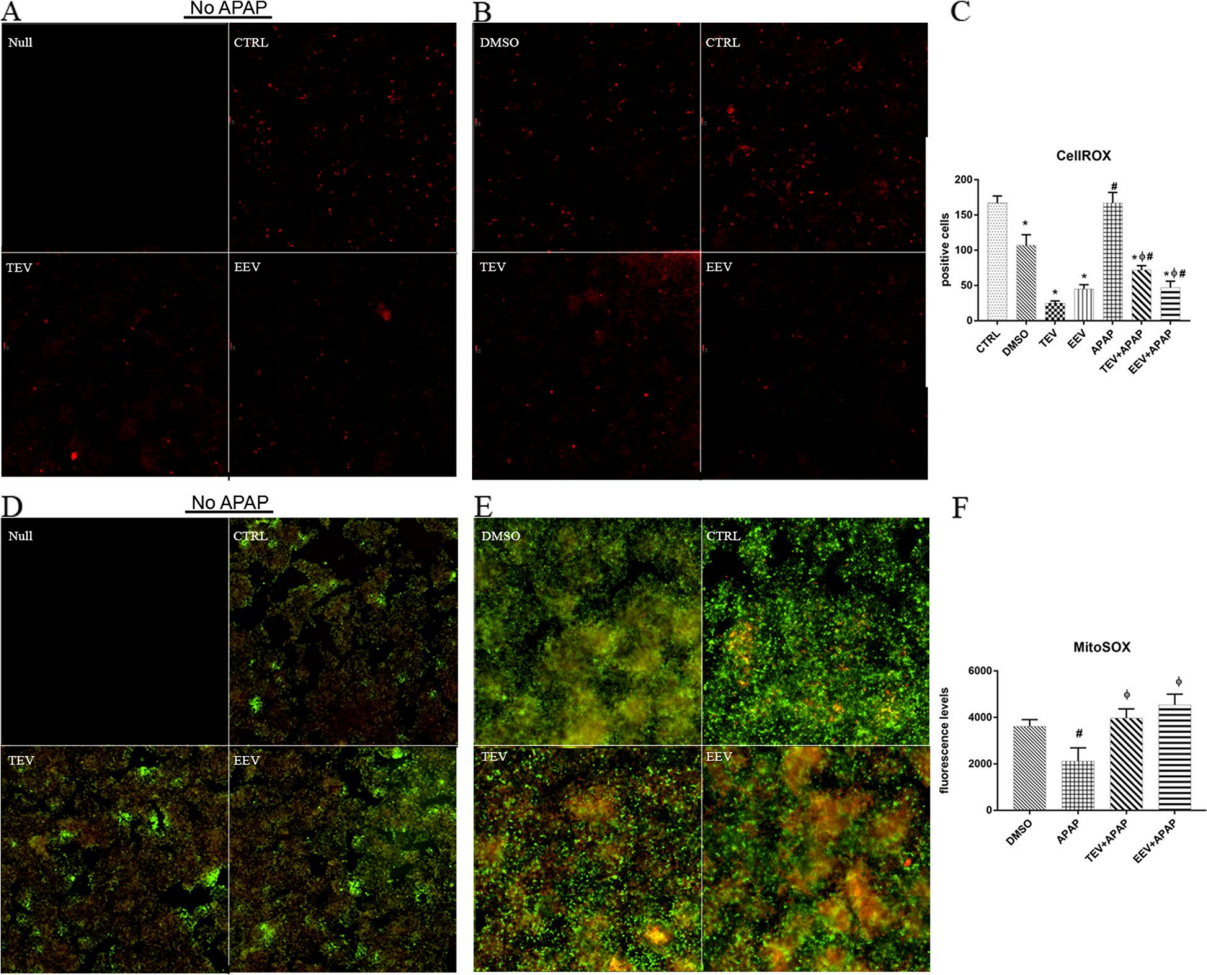
Live imaging of oxidative stress metabolic markers. Microphotographs taken stitching 4 images of HepG2 cells cultured on a 48-well plate of a live imaging immunofluorescence, representative for 3 experiments. Without APAP (panel **A** and **D**): no staining (Null), control cells (CTRL), TEV- and EEV-treated cells. With APAP (panel **B** and **E**): vehicle-treated cells (DMSO), control cells (CTRL), TEV- and EEV-treated cells. (A,B) CellROX staining (red) on HepG2 cells showing level of hydrogen peroxides in the cytoplasm (image size: 1560 × 1560 μm). **C** Diagram showing number of CellROX positive cells in CTRL, TEV and EEV-treated cells with or without 24 h APAP (or vehicle) treatment. After fluorescence background subtraction, positive cells in the microphotographs are counted. **D**, **E** MitoSOX staining (red) on HepG2 cells. MitoTracker counterstaining (green) showing basal mitochondrial ROS activity (image size: 1145 × 1145 μm). (F) Diagram showing fluorescence levels of MitoSOX positive cells in CTRL, TEV and EEV-treated cells with 24 h APAP (or vehicle) treatment. After fluorescence background subtraction, MitoSOX fluorescence levels were evaluated in positive counted cells. For all panels, data are mean ± SD with *n* = 3 per group. *, *p* < 0.05 vs CTRL; Φ, *p* < 0.05 vs APAP treatment; #, *p* < 0.05 vs vehicle (DMSO)

Respect to reactive oxygen species (ROS) production, we observed that in absence, as well as in presence of APAP, cell hydrogen peroxides detected by the CellROX reagent (Fig. 5A-C) were higher in control cells, if compared to cells treated with EEV and TEV fractions. On the contrary, intriguingly, baseline levels of MitoSOX (Fig. 5D-F), staining specifically for mitochondrial superoxide, were detectable even in absence of APAP, with a similar number of cells in all the samples (Fig. 5D). However, after APAP treatment, fluorescence levels rapidly increased in TEV and EEV fraction-treated cells, while drastically decreasing, being at least even absent, in APAP-treated control ones.

## Discussion

It is well documented that exosomes, endosomal derived small membrane vesicles, have a prominent role as mediators in cell-to-cell communication, influencing a large number of pathways in the recipient cells [15]. EV involvement in physiological and pathological processes paves the way to investigate their potential in clinical applications. Effects of EVs on recipient cells seem to be largely dependent on viability and health state of donor cells and, as a result of their origin, they may mediate totally different effects in recipient cells. For instance, exosomes derived from rat bone marrow mesenchymal stem cells were able to confer anti-apoptotic and/or prosurvival, as well as antioxidative effects, in in vitro models of liver injury [4]. In contrast, exosomes released from mice with APAP-induced DILI promoted death associated pathways in recipient cells, resulting in decreased cell viability [16].

As shown by studies cited herein, as well as by several others, exosomes have the ability to mediate crosstalk between different animal species. In the present work, we desired to explore as exosomes derived from an animal bred for food purposes could have an effect on human cells. In particular, we tested the effects of EVs derived from swine liver on liver-derived human cells, to explore whether the use of organ derivatives could be sustained for application as dietary supplements, also in so-called ‘functional foods’.

In this context, we decided to isolate EVs, exosomes in particular, with a polymer based separation, and a further purification with antibody-conjugated magnetic beads was also performed. Polymer, ExoQuick specifically, provides a low cost but highly efficient method to isolate exosomes. The disadvantage of this procedure is a possible contamination of samples with protein complexes, lipoproteins as well as other particles [17]. To circumvent this challenge, and improve EV purity, total fraction (TEV) was enriched (EEV) using magnetic beads coupled with human Rab5, that shares a high degree of homology with porcine one. It is well known that EVs devoid of the transmembrane protein used for selective exosome isolation exist and have functional activity [18], consequently both fractions, TEV and EEV, were used to perform experiments and comparison was particularly worthy of interest. As described in the Results section, both fractions are able to be up-taken by HepG2, in a couple of hours. However, only TEV fractions had an impact on cell growth in physiological conditions, significantly increasing the cell number, as demonstrated by results from both analyses, MTT assay and covered area; in contrast, EEV fraction effect was not detectable. Despite this, Ki67 overexpression in EEV and TEV fraction-treated cells was observable 48 h after EV administration, and confirmed that the fractions acted as a stimulus to cell growth rate improvement. Such different behaviour of the two fractions, led us to further explore and compare the TEV and EEV actions on the investigated cell population.

Under stressful conditions, after APAP administration, analysis of the covered area ratio confirmed a strong effect of the TEV fraction, although a protective outcome appeared to be mediated to a remarkably lesser extent also by EEV, as further inferred from MTT analysis. Taken together, our data suggest that EEV and TEV fractions may have significantly affected cellular metabolism, or at least the mitochondrial one, as well as the growth potential.

To better understand the mechanisms underpinning the identified protective effects, we explored different pathways. As reported in several studies, APAP induces apoptosis by increasing the cleavage of Caspase-3 and, as a consequence, raising c-PARP. Here, we show that EV pretreatment inhibited these pathways in a different manner: cells treated with the TEV fraction had a significant decrease in both cleaved proteins, whereas only a decrease in c-PARP was detectable in EEV fraction-treated cells.

Dissection of EV-mediated activation of p53 and p21, further provided evidence for a differential patterning elicited by TEV and EEV preconditioning in APAP-exposed cells. Consonant with the capability of TEVs to inhibit both c-Casp3 and c-PARP in APAP-treated cells, TEV preconditioning resulted more effective in relieving HepG2 cells from APAP-mediated inhibition of both p-p53 and p21 expression, while EEV pretreatment was only effective in counteracting the inhibitory action of APAP on p-p53 expression. Compounding the complexity of EV patterning, in the experiments conducted in the absence of APAP, TEV fraction by itself was less effective than EEV, being only able to upregulate p21 expression, while failing to affect p-p53. To date, the molecular interplay at the basis of TEV and EEV signaling still remains mostly enigmatic. The possibility that our observations may reflect multifaceted regulatory mechanisms at the gene, and protein expression level, as well as in post-translational modification remains to be explored and is the subject for our future investigations. It is known that p53 responds to cellular stress with a rapid phosphorylation and otherwise post-translational modifications that lead to its activation and stabilization [19]. P21 is a p53 effector, and its activation exerts an anti-apoptotic activity, also through Caspase-3 inhibition [20]. To this end, the capability of EV preconditioning to counteract the decline in APAP-induced p21, may be viewed as a synergistic pro-survival action to that exerted by TEVs and EEVs on p-p53. The capability of TEVs and EEVs to counteract APAP-induced downregulation of p27, while promoting its inhibition by themselves, may be considered as an additional circuitry opposing the apoptotic action of this hepatotoxic agent. In fact, although p27 (like p21) is regarded as a universal inhibitor of cell cycle progression, it can exert remarkable anti-apoptotic effects. Multiple evidence shows that apoptosis is fashioned in the G1 phase. P27 as a CDK/cyclin modulator provides regulation of apoptosis by interfering with the activity of these molecules. In particular, previous studies have demonstrated that apoptosis can occur when p27 levels are reduced, and that elevated p27 protects cells from an apoptotic fate by keeping CDK2 inactive. Nevertheless, other studies found that p27 may favor apoptosis, but mainly in highly invasive malignant cells [21]. The exact molecular mechanisms finely tuning the observed effects of EV preconditioning on p27 remain to be established, and their clarification must await more complex molecular and functional approaches.

Worthy of consideration, p53 could associate with JNK, inhibiting its activity, preventing JNK mitochondrial mediated cell death and caspase activation [22]. As a consequence of the non-specific binding of generated reactive metabolites to different mitochondrial proteins, a massive mitochondrial dysfunction arises, leading to ATP depletion, overproduction of ROS, JNK activation, and massive hepatocellular necrosis [23, 24]. Moreover, even if ER stress induced by APAP might also be a secondary consequence of mitochondrial dysfunction, it is demonstrated by several authors that, after APAP overdose, reactive metabolites generated by oxidation could trigger unfolded protein response (UPR) pathway [25]. After 24 h since APAP administration, we didn’t find a real impairment in protein involved in response to ROS. As described, APAP-treated cells displayed a slight increase in ROS production, but without noticeable overexpression of p-JNK, p-c-Jun or BiP protein. On the contrary, an increase in p-c-Jun and BiP was found in TEV fraction-treated cells, again highlighting a differential response to EVs, with enhanced effectiveness of TEV versus EEV preconditioning.

As shown by CellROX experiments, EVs downregulated cellular ROS production, in the presence, as well as in the absence of APAP. It is well known that ROS are strongly involved in normal physiological functions, such as cell cycle progression and differentiation, but their unbalance can lead to a deleterious oxidative stress [26]. CellROX analysis underlined the ability of EVs to downregulate cellular ROS amount, in cells treated or not with APAP. Intriguingly, an opposite result was obtained concerning mitochondrial ROS, through MitoSOX staining. These apparently contradictory data about ROS amount in different cell compartments, in fact, emphasize as EVs exercise a prominent effect on mitochondria. Indeed, enhanced mitochondrial activity was detected with MTT assay and confirmed by BiP increase. The molecular chaperone BiP is usually activated during ER stress, upon ROS accumulation in particular, in order to maintain proteins in a folding-competent state, until ER returns to a less oxidized state. BiP, in this context, may be considered as a direct sensor of ROS, as demonstrated in different types of cells [27]. Mitochondrial ROS have roles in several cell processes, including apoptosis and UPR and are not just dangerous molecules [28]*.*

## Conclusion

On the whole, the present study reveals the feasibility of using a farm animal food derivative of liver origin as a supplier of EVs capable to act on human hepatic cells, and afford remarkable protection against apoptotic and oxidative stress injuries. The current observations may pave the way to the development of nutraceuticals based upon the chance of delivering exosomal fractions, finely regulating essential determinants of cellular homeostasis and health.

## Supplementary Information

**Additional file 1.** Correlation of covered area with cells versus subsequent dilution of HepG2 cells.

**Additional file 2.** Quantification of ng/ml of CD81 positive vesicles after extraction and positive selection with Rab51.

**Additional file 3.** Uptake of EEV fraction in HepG2 cells after 24 h.

**Additional file 4.** Uptake of TEV and EEV fraction in HepG2 cells after 30 min

**Additional file 5.** Original images of full-length blots

**Additional file 6.** Western blot analysis of proteins involved in hepatic metabolism, adipogenesis and pathological cellular steatosis

## Data Availability

The datasets used and analyzed during the current study are available from the corresponding author.

## Abbreviations

EV(s): Extracellular vesicle(s)
MVBs: Multivesicular bodies
DILI: Drug induced liver injury
APAP: Acetaminophen
FBS: Foetal bovine serum
TEV(s): Total extracellular vesicle(s)
EEV(s): Enriched extracellular vesicle(s)
MTT: [3-(4,5-dimethylthiazol-2-yl)-2,5-diphenyltetrazolium bromide]
DMSO: Dimethylsulfoxide
M-PER: Mammalian Protein Extraction Reagent
HRP: Horseradish peroxidase
RT: Room temperature
BSA: Bovine serum albumin
JNK: c-Jun N-terminal kinase
ER: Endoplasmic reticulum
ROS: Reactive oxygen species
UPR: Unfolded protein response
