# Supplementary material for: Protective effects of exosomes derived from lyophilized porcine liver against acetaminophen damage on HepG2 cells
Source: BMC Complement Med Ther. 2021 Dec 18;21:299. doi: 10.1186/s12906-021-03476-y (PMC8684611; doi:10.1186/s12906-021-03476-y)
Supplement: Supplementary file 1 — Additional file 1. Correlation of covered area with cells versus subsequent dilution of HepG2 cells. [file 12906_2021_3476_MOESM1_ESM.pdf]

Additional file 1

Correlation of covered area with cells versus subsequent dilution of HepG2 cells.

In order to verify if the cell count using the covered\uncovered area is suitable and reliable, Hepg2 cells dilutions (from 100% to 6,6%) were seeded in the multiwell. After the overnight incubation, the area covered and an MTT analysis were performed. The linearity is shown since the Rsquare is 0.99 and 0.98 respectively.

(A) Area covered versus cell dilution analysis performed 16 hours after the seeding.

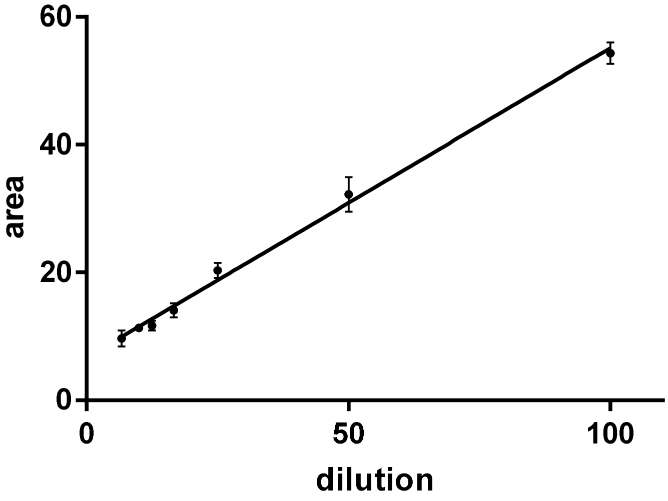

Best-fit values ± SE	
Slope	0.4845 ± 0.01378
Y-intercept	6.671 ± 0.6095
X-intercept	-13.77
1/slope	2.064
95% Confidence Intervals	
Slope	0.4491 to 0.5199
Y-intercept	5.104 to 8.237
X-intercept	-18.04 to -9.979
Goodness of Fit	
R square	0.996
Sy.x	1.131
Is slope significantly non-zero?	
F	1237
DFn, DFd	1, 5
P value	<0.0001
Deviation from zero?	Significant
Equation	Y = 0.4845*X + 6.671

(B) MTT absorbance versus cell dilution performed 16 hours after the seeding.

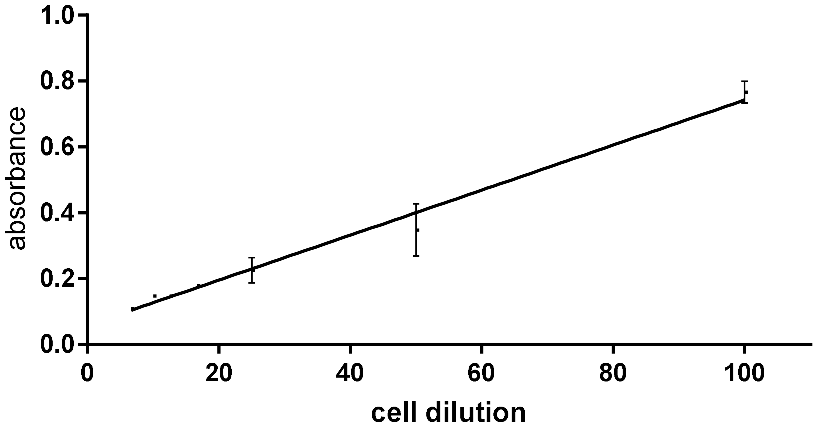

Best-fit values ± SE	
Slope	0.006841 ± 0.0003379
Y-intercept	0.05841 ± 0.01495
X-intercept	-8.538
1/slope	146.2
95% Confidence Intervals	
Slope	0.005973 to 0.00771
Y-intercept	0.01998 to 0.09684
X-intercept	-15.72 to -2.674
Goodness of Fit	
R square	0.988
Sy.x	0.02773
Is slope significantly non-zero?	
F	410
DFn, DFd	1, 5
P value	<0.0001
Deviation from zero?	Significant
Equation	Y = 0.006841*X + 0.05841
