# Supplementary material for: Protective effects of exosomes derived from lyophilized porcine liver against acetaminophen damage on HepG2 cells
Source: BMC Complement Med Ther. 2021 Dec 18;21:299. doi: 10.1186/s12906-021-03476-y (PMC8684611; doi:10.1186/s12906-021-03476-y)

Additional file 2

Quantification of ng/ml of CD81 positive vesicles after extraction and positive selection with Rab51.

In order to obtain a reliable quantification of the concentration of the vesicles inside the samples extracted, we used an ELISA based assay that quantify the CD81 present. The graph (A) shows the standard curve of the ELISA test, while the table shows exemplificative results of four EEV samples analysed.

In the graph (B) a standard samples of a known number of exosomes are tested on the same ELISA kit.

(A)

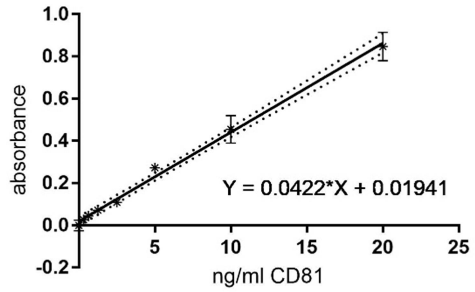

Sample number	absorbance	Mean	Mean-blank	ng/ml
1	0.097	0.098	0.025	0.15
1	0.100			
2	0.101	0.100	0.027	0.19
2	0.102			
3	0.98	0.100	0.027	0.19
3	0.97			
4	0.103	0.101	0.028	0.21
4	0.091			
blank	0,073	0,073		
blank	0,073			

(B)

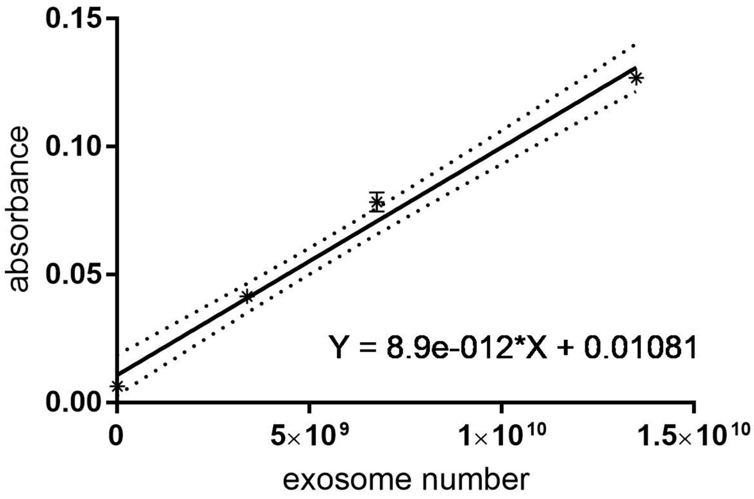

Supplement: Supplementary file 2 — Additional file 2. Quantification of ng/ml of CD81 positive vesicles after extraction and positive selection with Rab51. [file 12906_2021_3476_MOESM2_ESM.pdf]
