# Supplementary material for: Protective effects of exosomes derived from lyophilized porcine liver against acetaminophen damage on HepG2 cells
Source: BMC Complement Med Ther. 2021 Dec 18;21:299. doi: 10.1186/s12906-021-03476-y (PMC8684611; doi:10.1186/s12906-021-03476-y)

Additional file 3

Uptake of EEV fraction in HepG2 cells after 24 hours.

(A) BODIPYTM TR Ceramide labelled (red) EEVs showing uptake in HepG2 cells after 24h. Shown here are microphotographs of HepG2 cells cultured on a 48-well plate – with the two channel (brightfield and fluorescence) together and separated; The localization inside the cells, outside the nucleus, and primary around the nucleus, suggest the endoplasmic reticulum incorporation of the fluorophore.

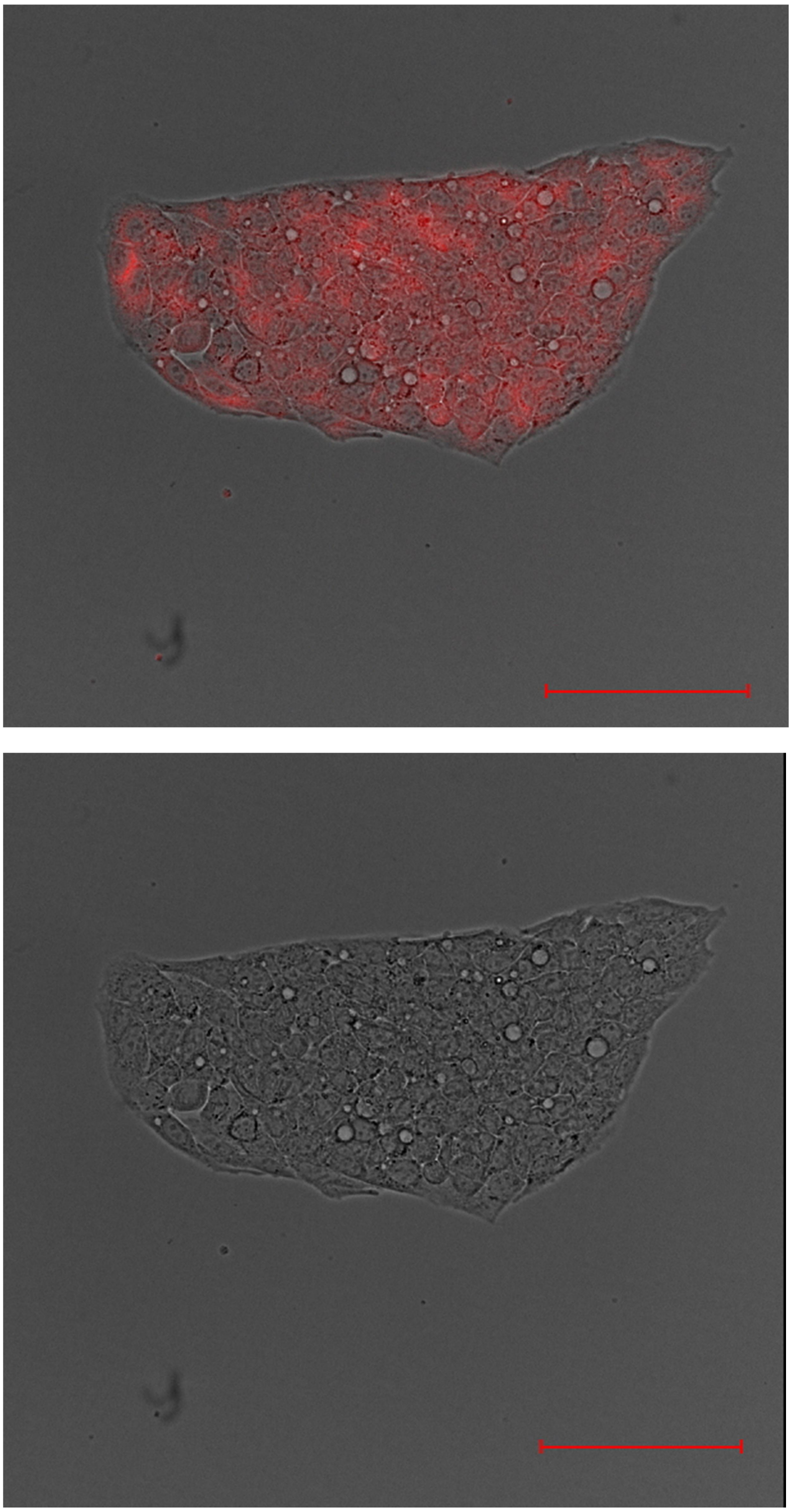

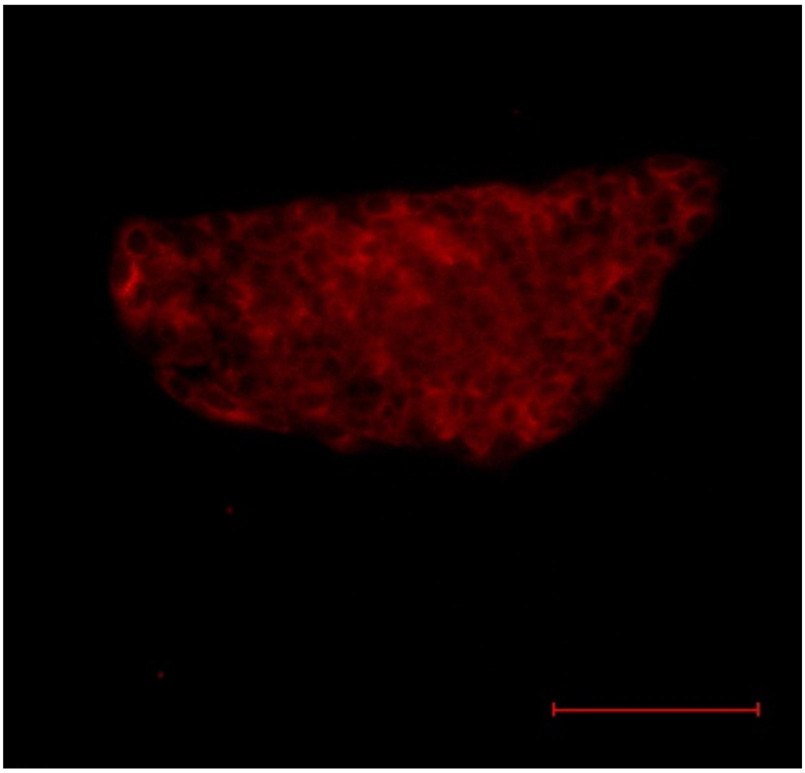

(B) BODIPYTM TR Ceramide labelled (red) EEVs showing uptake in Mesenchymal stem cells after 24h. Shown here are microphotographs of hMSCs cells cultured on a 48-well plate. The localization inside the cells, outside the nucleus, and primary around the nucleus, suggest the endoplasmic reticulum incorporation of the fluorophore.

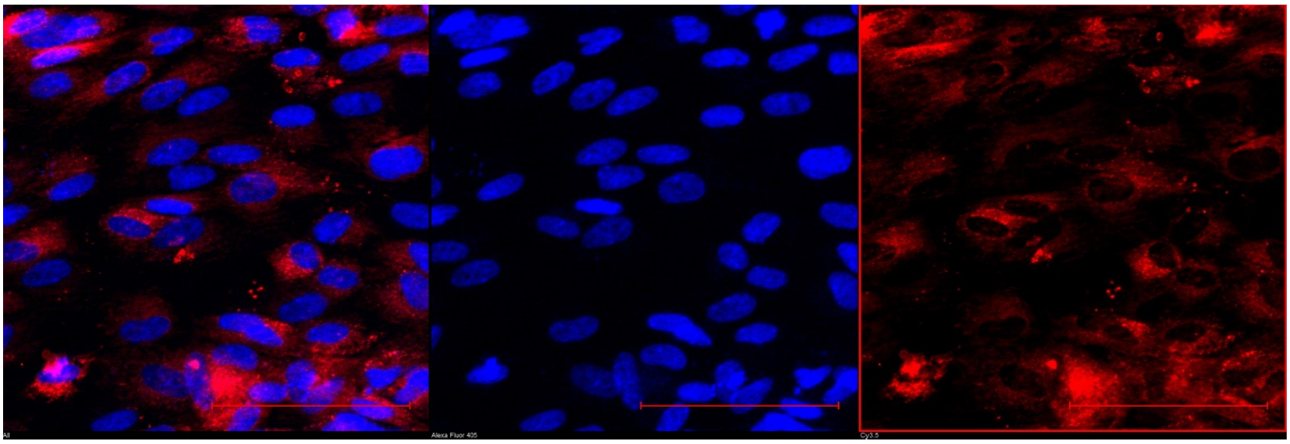

Supplement: Supplementary file 3 — Additional file 3. Uptake of EEV fraction in HepG2 cells after 24 h. [file 12906_2021_3476_MOESM3_ESM.pdf]
