# Supplementary material for: Protective effects of exosomes derived from lyophilized porcine liver against acetaminophen damage on HepG2 cells
Source: BMC Complement Med Ther. 2021 Dec 18;21:299. doi: 10.1186/s12906-021-03476-y (PMC8684611; doi:10.1186/s12906-021-03476-y)

Additional file 4

Uptake of TEV and EEV fraction in HepG2 cells after 30 minutes (A) BODIPY^{TM} TR Ceramide labelled (red) TEVs and EEVs showing uptake in HepG2 cells. Shown here are microphotographs (384 x 384 µm) taken stitching 4 images of HepG2 cells cultured on a 48-well plate; representative for 5 experiments.

EEV Bodipy
30 minutes
Overexposed
acquisition

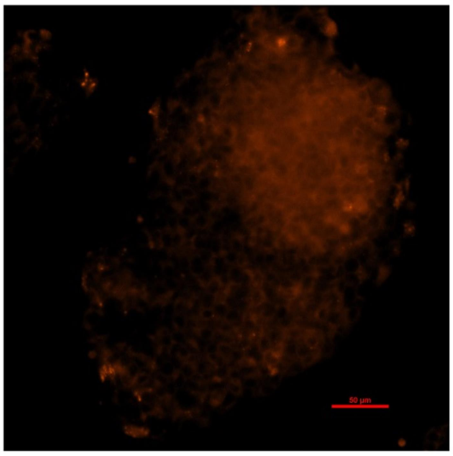

TEV Bodipy
30 minutes
Overexposed
acquisition

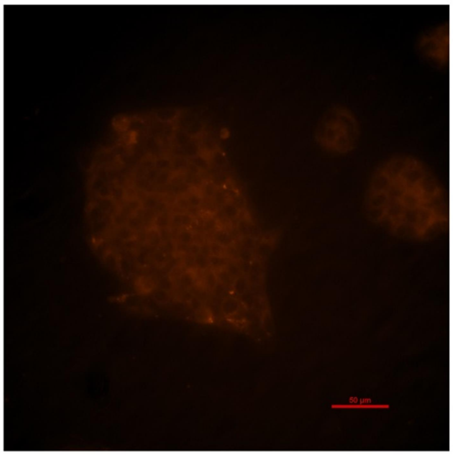

Supplement: Supplementary file 4 — Additional file 4. Uptake of TEV and EEV fraction in HepG2 cells after 30 min [file 12906_2021_3476_MOESM4_ESM.pdf]
