# Supplementary figures and images for: Protective effects of exosomes derived from lyophilized porcine liver against acetaminophen damage on HepG2 cells
Source: BMC Complement Med Ther. 2021 Dec 18;21:299. doi: 10.1186/s12906-021-03476-y (PMC8684611; doi:10.1186/s12906-021-03476-y)

# Additional file 5

Original images of full-length blots

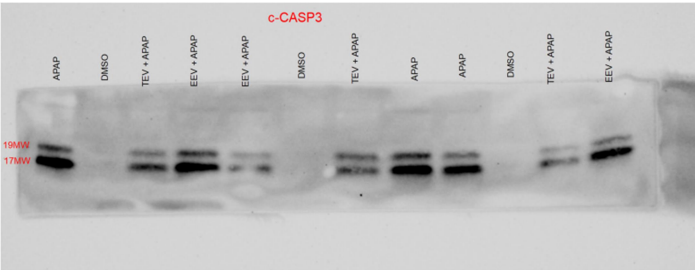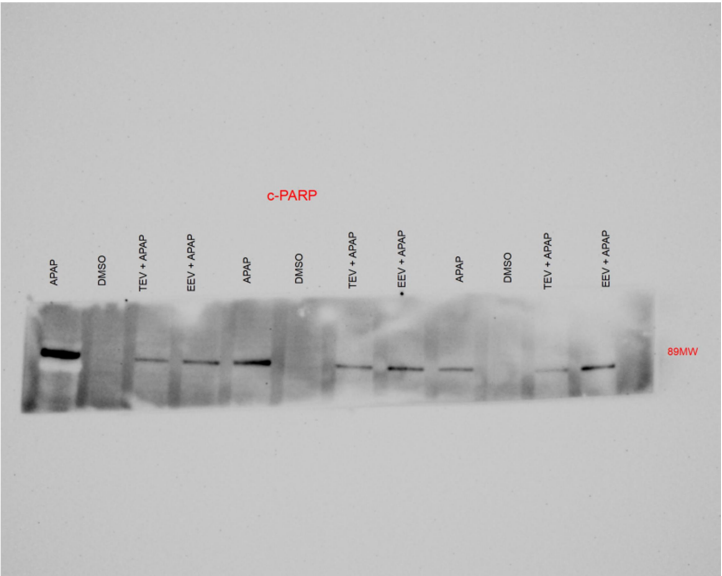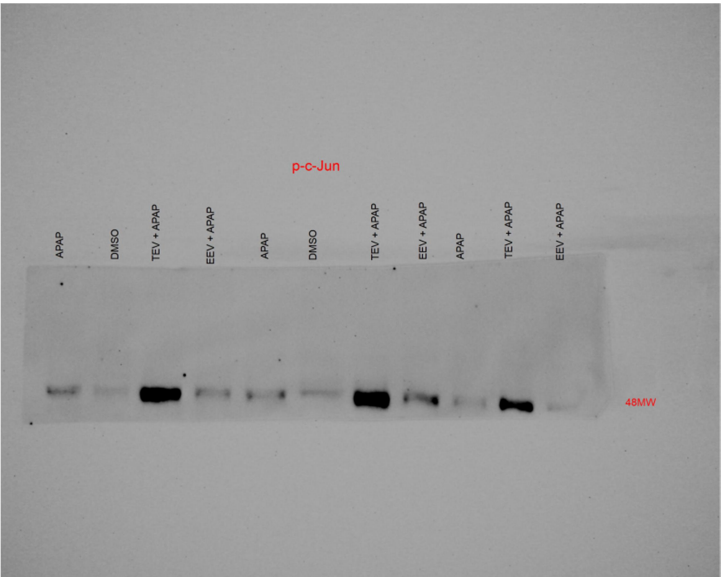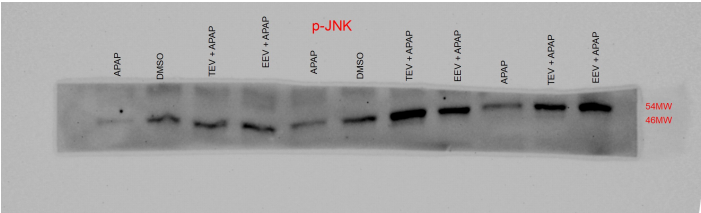

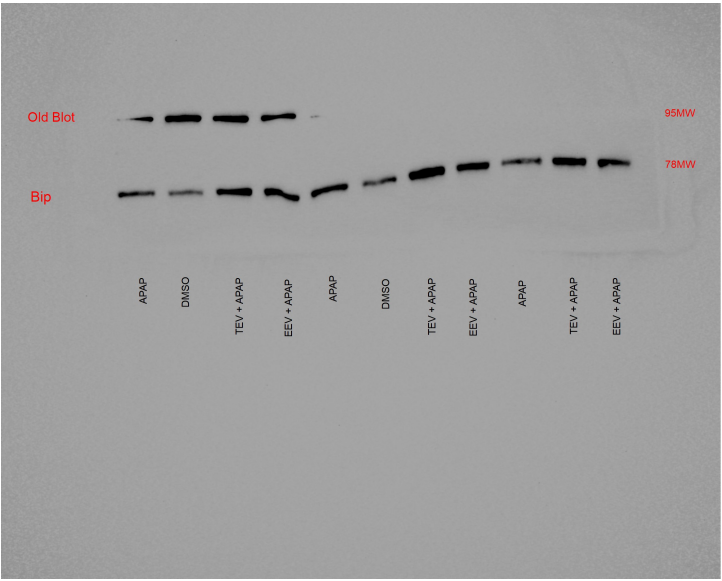

Supplement: Supplementary file 5 — Additional file 5. Original images of full-length blots [file 12906_2021_3476_MOESM5_ESM.pdf]
