# Supplementary material for: Protective effects of exosomes derived from lyophilized porcine liver against acetaminophen damage on HepG2 cells
Source: BMC Complement Med Ther. 2021 Dec 18;21:299. doi: 10.1186/s12906-021-03476-y (PMC8684611; doi:10.1186/s12906-021-03476-y)

Additional file 6

Western blot analysis of proteins involved in hepatic metabolism, adipogenesis and pathological cellular steatosis.

Antibodies and dilutions. Primary antibodies used for Western analysis (1:2,000 dilution) were all purchased by Cell Signaling Technology: mammalian target of rapamycin (mTOR, #2972), fatty acid synthase (FASN, #3180), acetyl-CoA carboxylase (ACC, #3676), fatty acid binding protein 4 (FABP4, #3544), eukaryotic initiation factor 2 (Eif2a, #5324). As secondary antibody, 1:10,000 anti-rabbit IgG, HRP-linked Antibody (#7074) (Cell Signaling Technology) was used.

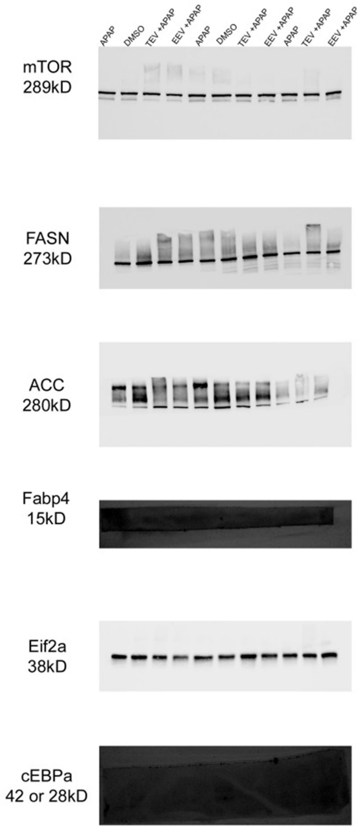

Supplement: Supplementary file 6 — Additional file 6. Western blot analysis of proteins involved in hepatic metabolism, adipogenesis and pathological cellular steatosis [file 12906_2021_3476_MOESM6_ESM.pdf]
